# Sumoylation of CCAAT‐enhancer‐binding protein α inhibits lung differentiation in Bronchopulmonary Dysplasia model rats

**DOI:** 10.1111/jcmm.15310

**Published:** 2020-05-04

**Authors:** Yue Zhu, Xiaoqing Chen, Lanlan Mi, Qiuxia Wang, Haitao Zhu, Huimin Ju, Hongyan Lu

**Affiliations:** ^1^ Department of Pediatrics Affiliated Hospital of Jiangsu University Zhenjiang China; ^2^ Department of Pediatrics The First Affiliated Hospital of Nanjing Medical University Nanjing China

**Keywords:** bronchopulmonary dysplasia, CCAAT enhancer binding protein alpha, differentiation, pulmonary surfactant, rats, sumoylation

## Abstract

Bronchopulmonary dysplasia (BPD) is a major cause of mortality and morbidity in premature infants, characterized by alveolar simplification, surfactant deficiency, and respiratory distress. In the present study, we have investigated the functional roles of sumoylated CCAAT/enhancer binding protein alpha (C/EBPα) in the BPD rat model. A significant increase in small ubiquitin‐like modifier 1 (SUMO1) and sumoylated C/EBPα protein levels were observed in BPD rats, and the levels of the sumoylated C/EBPα were associated with the pulmonary surfactant proteins (SPs). In order to confirm the role of sumoylated C/EBPα in BPD rats, SUMO1 was knocked down by lentiviral transfection of neonatal rat lungs with SUMO1‐RNAi‐LV. We found that the expression of C/EBPα and surfactant proteins increased following SUMO1 knockdown. Furthermore, the relatively low decrease in the levels of C/EBPα sumoylation was correlated with reduced glycogen consumption. Besides, co‐immunoprecipitation assays revealed that sumoylation is involved in the regulation of the interaction between C/EBPα and TGFβ2 in the lung. In conclusion, our findings indicate that sumoylation may act as a negative regulator of the C/EBPα‐mediated transactivation in BPD rats.

## 1 INTRODUCTION

Bronchopulmonary dysplasia (BPD) is a common complication of premature infants.^1^, ^2^ A recent study has demonstrated that gene regulation is implicated in lung development has aroused a great interest with particular focus on the expression of CCAAT‐enhancer‐binding proteins (C/EBPs).

C/EBPs are a family of basic region/leucine zipper (bZIP) transcription factors, and C/EBPα participates in the regulation of lung differentiation and stimulates pulmonary gene expression patterns/characteristics in the mature differentiated epithelium.^3^, ^4^ Sumoylation is a process of attaching small ubiquitin‐like modifiers (SUMOs) to protein substrates at specific lysine residues. Recently, C/EBPα was reported to be modified post‐transitionally by SUMO1 at a lysine residue within its attenuator domain motif that causes an inhibitory effect on its transcriptional activity.^5^, ^6^, ^7^, ^8^ Our previous study indicated that sumoylated C/EBPα may be related to lung differentiation and alveolar surfactant protein expression during lung development.^9^ However, whether pulmonary differentiation disorder is related to imbalance of sumoylation of C/EBPα in BPD still remains unclear. Here, we explored the functional roles of sumoylation C/EBPα and its association between lung differentiation and confirmed the regulatory effect of sumoylation in the C/EBPα‐mediated transactivation by using lentivirus vector‐mediated *SUMO1* siRNA in the rat BPD model.

## 2 MATERIALS AND METHODS

### 2.1 Establishment of the BPD rat model

Sprague‐Dawley rats (SD, 90‐100 days old, 250‐300 g) were provided by the Animal Center of Jiangsu University. All animal experiments were approved by the laboratory of the Animal Ethics Committee of Jiangsu University. The BPD animal model was constructed as previously described.^10^ Three to five newborn rats per group were dissected, and their lungs were removed at the postnatal day 7 and day 14 (P7 and P14) for further study.

### 2.2 Administration of Lentivector to Neonatal Rat Lung

In order to confirm the role of sumoylated C/EBPα in BPD rats, *SUMO1* was silenced by lentiviral transfection of neonatal rat lungs with *SUMO1‐*RNAi‐LV. Detailed protocol is presented in supporting information.

### 2.3 RT‐PCR, Western blot and Co‐immunoprecipitation (Co‐IP) assay

We examined the mRNA or protein expression of SUMO1, C/EBPα, TGβ2, SPs and β‐actin by Western blot and RT‐PCR as described previously.^9^, ^11^ The primer sequences used in this study are shown in Table S1. Co‐IP assay was used to detect the level of sumoylated C/EBPα and the interaction of C/EBPα with TGβ2. More detailed protocol and antibody details are presented in supporting information.

### 2.4 Periodic acid‐Schiff (PAS) staining and Immunofluorescence

Tissues sections were stained with PAS staining for analysing the content of glycogen as described earlier.^9^ Double‐labelled immunofluorescent staining was used to detect the co‐localization of C/EBPα and TGFβ2. More detailed protocol and antibody details are presented in supporting information.

### 2.5 Statistical analysis

Values are presented as mean ± SD. Comparisons between two groups were performed by the independent samples t test, while comparisons among multiple groups were performed using a one‐way analysis of variance (ANOVA) with Tukey's multiple comparison post hoc test. Differences were considered statistically significant when *P* < .05.

## 3 RESULTS

### 3.1 Expression of SUMO1, sumoylated C/EBPα and SPs in BPD rats

**Figure 1:**
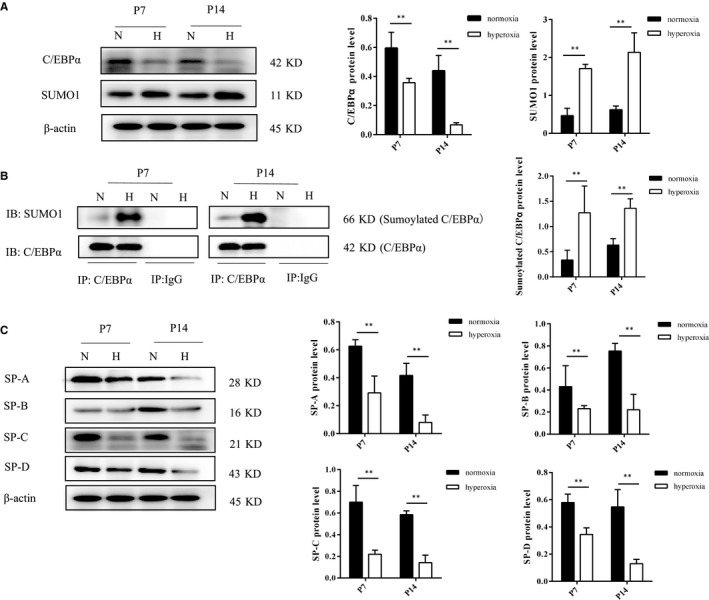
The sumoylation of C/EBPα and its relationship with pulmonary surfactant protein expression in BPD rat. Three neonatal rats per group were selected were killed from newborn rats at P7 and P14 and one sample repeated three times in our experiment; normoxia group: 21% O_2,_ hyperoxia group: 80%‐85% O_2_. The protein expression of SUMO1, C/EBPα and pulmonary surfactant proteins (SPs, including SP‐A, SP‐B, SP‐C and SP‐D) was detected by Western blot assays in the four groups. β‐Actin was used as the loading control (A and C). Co‐immunoprecipitation (Co‐IP); the levels of sumoylated C/EBPα were significantly increased during hyperoxia conditions (B). Values represent mean ± SD; ** *P* < .05 vs normoxia group

We observed low protein expression of SUMO1 in normoxia group, while high expression in the hyperoxia group at both P7 and P14. In contrast to the normoxia group, the hyperoxia group showed decreased expression of C/EBPα protein (Figure 1A). The Co‐IP results showed the presence of sumoylated C/EBPα protein in the lung samples (Figure S1). Further analysis revealed that the levels of C/EBPα sumoylation were low under normal lung development, while they significantly increased during hyperoxia conditions (Figure 1B). The levels of SP‐A, SP‐B, SP‐C and SP‐D proteins were significantly reduced under hyperoxia conditions (Figure 1C). Moreover, our data showed that sumoylation of C/EBPα is negatively correlated with the levels of C/EBPα and SPs. These findings suggested that sumoylation plays a key role in the regulation of C/EBPα expression and C/EBPα‐mediated lung differentiation.

### 3.2 *SUMO1* knockdown regulates SPs expression and glycogen content in BPD rats

**Figure 2:**
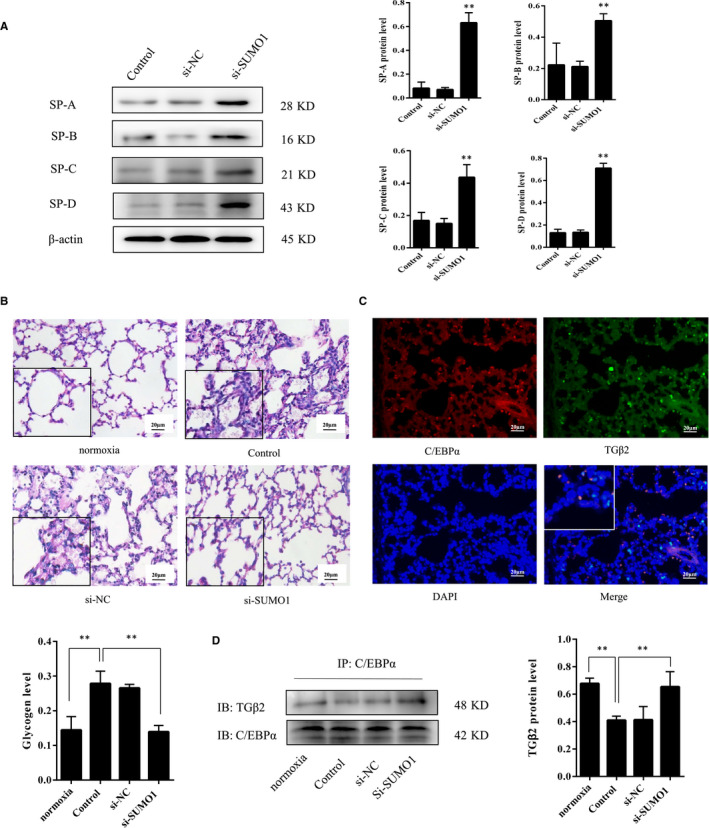
The changes of pulmonary surfactant protein expression, glycogen content and the interaction between C/EBPα and TGFβ2 following *SUMO1* knockdown in BPD rats. Three neonatal rats per group were selected were killed from newborn rats at P14 and one sample repeated three times in our experiment. Comparison of the expression levels of pulmonary surfactant proteins in the rat lungs among control, si‐NC and si‐SUMO1 groups (A). Periodic acid and Schiff (PAS) staining assay on rat lung tissues from the different groups (B). Immunofluorescence imaging; C/EBPα was partially co‐localized with TGFβ2 in the rat lung tissues (C). Co‐immunoprecipitation assay (Co‐IP); the levels of co‐immunoprecipitated C/EBPα and TGFβ2 expression in different groups (D). Scale bar = 20 µm; original magnifications: ×200; square frame magnification: ×400. Values are presented as mean ± SD; ** *P* < .05 vs control

Following *SUMO1* knockdown in the lungs of BPD rats, RT‐PCR and Western blot revealed a significant increase, respectively, in the mRNA and protein levels of C/EBPα (Figure S2A‐B). Analysis by immunoprecipitation after transfection with *SUMO1*‐RNAi‐LV (si‐SUMO1) demonstrated that the expression levels of C/EBPα sumoylation decreased in comparison with the other two groups (Figure S2C). The expression levels of SP‐A, SP‐B, SP‐C and SP‐D were increased to varying degrees and were negatively correlated with the levels of C/EBPα sumoylation after *SUMO1* knockdown (Figure 2A). As shown by PAS staining, the content of glycogen significantly increased in hyperoxia‐exposed rats and reduced after treatment with *SUMO1‐*RNAi‐LV (Figure 2B). These results demonstrate that *SUMO1* knockdown increases C/EBPα expression, SPs secretion and lung differentiation.

### 3.3 *SUMO1* knockdown affects the interaction of C/EBPα with TGFβ2 in BPD rats

TGFβ2 is a growth factor that inhibits differentiation of alveolar type II epithelial cells during lung maturation.^12^ Immunofluorescent staining showed that C/EBPα and TGFβ2 were partially co‐localized in the nucleus of lung tissues at P14 (Figure 2C). The Co‐IP results suggest that C/EBPα interacts with TGFβ2 in the rat lungs (Figure S3A). In addition, *SUMO1* knockdown increased the interaction of C/EBPα with TGFβ2 (Figure 2D). Taken together, these results reveal that sumoylation is involved in the regulation of the interaction of C/EBPα with TGFβ2 in the lung.

## 4 DISCUSSION

Our results show that hyperoxia induces the levels of C/EBPα sumoylation and reduces the levels of C/EBPα and SPs in BPD rats. In addition, *SUMO1* knockdown promotes the expression of C/EBPα, SPs as well as the content of glycogen. These findings suggest that SUMO modification may be involved in the regulation of C/EBPα‐mediated AECII differentiation and secretion. It has been previously shown that HDACs are involved in the transcriptional inhibition of sumoylated transcription factors and sumoylated C/EBPα interacts with HDAC3 to exert repressive activity.^13^ Sato et al^14^ have reported that the attachment of SUMO1 to C/EBPα inhibited the recruitment of the SWI/SNF complex to the albumin gene in hepatocytes, which consequently enhanced C/EBPα‐mediated auto‐regulation. Moreover, some sumoylated proteins can be recognized by E3 ubiquitin ligases which can recognize SUMO modification, and further degraded by ubiquitin pathway. Taken together, we assume that sumoylation may inhibit the transcriptional activity of C/EBPα and reduce the expression of C/EBPα by inhibiting C/EBPα auto‐regulation; or sumoylation of C/EBPα could be increasing the rate of protein degradation via sumoylation‐coupled ubiquitination.

Additionally, *SUMO1* knockdown promoted the interaction of C/EBPα with TGFβ2, suggesting that sumoylation is involved in the regulation of the interaction of C/EBPα with TGFβ2 in the lung. As previously reported, deletion of the *C/EBPα* gene causes increased expression of TGFβ2 in the lung.^15^ Thus, we speculate that SUMO1 is involved in the conformational transformation of C/EBPα following its modification, or directly occupied the binding sites of TGFB2 and C/EBPα. However, the mechanisms underlying this relationship are still unclear and warrant further research.

In conclusion, we provided reliable evidence that sumoylated C/EBPα participates in the pathogenesis of BPD and further demonstrated that sumoylation exhibits a negative effect on AECII differentiation and secretion during lung injury induced by hyperoxia in newborn rats. Our studies shed new insight into the role of protein sumoylation in lung development and may provide rationale for targeting the SUMO pathway for potential BPD therapy.

## CONFLICT OF INTEREST

The authors declare no competing interests.

## AUTHOR CONTRIBUTIONS

YZ performed the majority of the laboratory work, statistics and data analysis and participated in writing. LLM and QXW were involved in writing. HYL and CXQ conceived and designed the experiments. HTZ and HMJ participated in discussions and drafted the manuscript.

## Supporting information

Supplementary Material

## Data Availability

The data sets generated during and/or analysed during the current study are available from the corresponding author on reasonable request.
