# Supplementary material for: Sumoylation of CCAAT‐enhancer‐binding protein α inhibits lung differentiation in Bronchopulmonary Dysplasia model rats
Source: J Cell Mol Med. 2020 May 4;24(12):7067–71. doi: 10.1111/jcmm.15310 (PMC7299724; doi:10.1111/jcmm.15310)
Supplement: Supplementary file 1 — Supplementary Material [file JCMM-24-7067-s001.docx]

**SUPPORTING INFORMATION**

***1 MATERIALS AND METHODS***

**1.1 Ethics compliance**

The research was conducted in accordance with the Guide for Care and use of Laboratory Animals as adopted and promulgated by the United National Institutes of Health. All animal experimental protocols were reviewed and approved by the laboratory of the Animal Ethics Committee of Jiangsu University.

**1.2 Establishment of the BPD rat model**

Sprague-Dawley rats (SD, 90-100 days old, 250-300 g) were provided by the Animal Center of the Jiangsu University (Zhenjiang, China). The BPD animal model was constructed as previously described^1^. Newborn SD rats were randomly divided into two groups, and were exposed to hyperoxia (80-85% O_2_; hyperoxia group) and room air (21% O_2_; normoxia group) at the beginning of the day of their birth. Three to five newborn rats per group were dissected and their lungs were removed at the postnatal day 7 and day 14 (P7 and P14). The left lungs were fixed with 4% paraformaldehyde and the right lungs were stored at -80 ˚C.

**1.3 Administration of Lentivector to Neonatal Rat Lung**

Small interfering RNAs (siRNAs) were designed to target the nucleotide sequence of the rat *SUMO1* gene (NM001009672). The lentiviral solution containing the siRNAs against *SUMO1* (*SUMO1-*RNAi-LV) and the negative siRNA control (pGC FU-RNAi-NC-LV) were provided by the Shanghai GeneChem Co. (Shanghai, China). Newborn SD rats were randomized on postnatal day 1 into three groups: hyperoxia (control group); hyperoxia + *SUMO1-*RNAi-LV (si-SUMO1 group); and hyperoxia + pGC FU-RNAi-NC-LV (si-NC group). Lentiviral solution with *SUMO1-*RNAi-LV (5×10^8^ TU/ml) and pGC FU-RNAi-NC-LV (equal concentration) was administered intranasally on day 4 during hyperoxia exposure. The rats were exposed to isoflurane for 30 seconds to achieve mild anaesthesia, and the lentiviral solution was administered into the nasal passages using a microprojection syringe. Slight pressure was applied to the bottom of the jaw with the thumb to force the rats to inhale the solution through the nose. Gauldie et al.^2^ have shown that this approach results in a widespread distribution of the vector throughout the lungs. Lung tissues were harvested 10 days after lentivector injection. Three to five neonatal rats per group were selected for further assays and one sample was repeated three times in our experiment.

**1.4 RT-PCR assay**

Chopped rat lung tissues were treated with liquid nitrogen and grounded into powder. In accordance to the manufacturer’s instructions, RNA was extracted from the lung powder with TRIzol Reagent (Invitrogen, Carlsbad, USA). Total RNA was isolated by phenol-chloroform extraction and precipitated with isopropanol. The concentration of total RNA was measured by a spectrophotometer. The first strand of cDNA was synthesized from RNA samples in strict accordance with the instructions of the reverse transcription kit (Thermo Fisher Scientific, Sunnyvale, CA, USA). SUMO1 or C/EBPα amount of cDNA was used as a template for RT-PCR in each of the 3 replicated samples (Three neonatal rats were sacrificed in each group and one sample repeated three times in our experiment). Quantitative analysis was performed on the Roche Light Cycler Sequence Detection System (Roche, USA). The primer sequences used in this study are shown in Table S1. Each reaction was carried out three times. The relative expression levels of target genes were calculated using beta-actin as the internal control. The threshold cycle (Ct) values were determined and used to analyze gene expression using the 2^-△△CT^ quantification approach.

**1.5 Western blot assay**

The lung tissues were thawed on ice, washed with cold PBS, lysed in proper volume of cell lysis buffer containing protease inhibitor PMSF (1:100, Sigma, Darmstadt, Germany), and centrifuged at 12,000 g at 4 ˚C for 15 min; next, 5x loading buffer was added to the supernatant. Lysates were boiled for 8 min, then separated by 12% SDS-PAGE at a constant voltage of 70 V, and transferred to polyvinylidene difluoride (PVDF) membranes for 90 min under a constant current of 350 mA. The PVDF membranes were blocked with 5% non-fat milk in TBST containing 0.1% Tween at 37 ˚C for 1 h, and incubated at 4 ˚C overnight with the following primary antibodies: anti-C/EBPα (sc9314, 1:200, Santa Cruz, USA), anti-SUMO1(ab32058, 1:1000, Abcam, UK), anti-SP-A (LS-C357574, 1:500, LifeSpan BioSciences, USA), anti-SP-B (sc7704, 1:200, Santa Cruz, USA), anti-SP-C (sc13979, 1:200, Santa Cruz, USA), anti-SP-D (bs-1583R, 1:500, BIOSS, China), anti-TGβ2 (ab113670, 1:1000, Abcam, UK) and anti-β-actin (#3700, 1:1000, CST, USA). The membranes were washed three times with TBST and then incubated with biotinylated secondary antibodies (FMS-MS01, FMS-Rb01, FMS-Gt01, 1:5000, Fcmacs Biotech, China) for 1 h at 37 ˚C. Eventually, the immunoreactive bands were visualized by FluorChem FC3 chemiluminescence (ProteinSimple, San Jose, CA, USA). Protein contents were densitometrically calculated using the LANE 1D software (Sage, Beijing, China), and the relative protein expression levels were calculated as target protein/β-actin. Three neonatal rats were sacrificed in each group and one sample repeated three times in our experiment.

**1.6 Co-Immunoprecipitation assay**

In order to detect the expression of sumoylated C/EBPα, co-immunoprecipitation assay was carried out. Then, we normalized WB to CEBPa levels post IP, which can compare the variation of SUMO modification and C/EBPa binding reasonably. The lung tissues were thawed on ice, washed with cold PBS, and lysed in NP40 buffer supplemented with protease inhibitor PMSF(1:100) and N-ethylmaleimide (NEM) (1:200). Lysates were incubated with anti-C/EBPα (1:100) or control IgG (1:100) antibody at 4 ˚C for 2 h under constant rotation, and bound to Protein A/G PLUS-Agarose beads (Santa Cruz, USA). Following, the mixture was incubated overnight at 4 ˚C and washed at least three times with the lysis buffer. Proteins were mixed with 5x loading buffer containing β-dithiothreitol, separated by 12% SDS-PAGE, transferred to PVDF membranes, and immunoblotted with anti-SUMO1 and anti-C/EBPα antibodies. To detect the interaction of C/EBPα and TGβ2, immunoprecipitation was performed as described above and the immunoprecipitated proteins were blotted with anti-TGβ2 and anti-C/EBPα antibodies. A total of 3 samples/group were analyzed and one sample repeated three times in our experiment.

**1.7 Periodic acid-Schiff (PAS) staining**

As the lung matures after birth, the intracellular glycogen is gradually consumed and transformed into pulmonary surfactant phospholipids or lamellar bodies to maintain lung function. The content of glycogen can reflect the development of lung tissues and can be used as a reference indicator^3^. The lung tissues were fixed with 4% paraformaldehyde at 4 ˚C overnight, washed with PBS and processed into paraffin blocks. Tissues sections were stained with periodic acid-Schiff (PAS) reagent (Beijing Solarbio Science & Technology Co, Beijing, China) for analyzing the content of glycogen. Positive glycogen staining was visualized in red or purple color. The sections were observed under a light microscope and the images were analyzed by Image-Pro Plus III (Media Cybernetics, Inc., Rockville, MD, USA) to obtain the mean gray value. A total of 3 samples/group were analyzed and one sample repeated three times in our experiment.

**1.8 Immunofluorescence**

Double-labeled immunofluorescent staining was used to detect the co-localization of C/EBPα and TGFβ2. After deparaffinization and antigen retrieval, the lung tissue sections were blocked with 5% serum in PBS for 30 min and incubated with a mixture of anti-C/EBPα (1:100) and anti-TGFβ2 (1:100) antibodies at 4 ˚C overnight. The sections were washed three times with PBS and incubated with secondary PE-conjugated antibody (1:500, bs-0294D-PE, BIOSS, China) and FITC-conjugated antibody (1:500, bs-0295D-FITC, BIOSS, China). Fluorescent staining of the lung tissues was visualized under an inverted ﬂuorescent microscope. Three neonatal rats were sacrificed in each group and one sample repeated three times in our experiment.

**1.9 Statistical analysis**

Three to five newborn rats per group were dissected and one sample repeated three times in our experiment. Values are presented as mean ± SD. All data were analyzed by the SPSS 19.0 statistical software. Comparisons between two groups were performed by the Independent Samples t-test, while comparisons among multiple groups were performed using a one-way analysis of variance (ANOVA) with Tukey’s multiple comparison post hoc test. Differences were considered statistically significant when *P* < 0.05.

**REFERENCES**

1. Warner BB, Stuart LA, Papes RA, et al. Functional and pathological effects of prolonged hyperoxia in neonatal mice. *American Journal of Physiology*. 1998; 275: 110-7.

2. Gauldie J, Galt T, Bonniaud P, et al. Transfer of the Active Form of Transforming Growth Factor-β1 Gene to Newborn Rat Lung Induces Changes Consistent with Bronchopulmonary Dysplasia. *American Journal of Pathology*. 2003; 163: 2575-84.

3. Ridsdale R, Post M. Surfactant lipid synthesis and lamellar body formation in glycogen-laden type II cells. *Am J Physiol Lung Cell Mol Physiol*. 2004; 287: 743-751.

***2 ADDITIONAL RESULTS***

**Supplementary Table S1**:

Table S1 Primer pairs used for qRT-PCR.

| Gene |  | Primer Sequence (5'-3') |
| --- | --- | --- |
| C/EBPα | F | 5′-gccaagaagtcggtggataa-3′ |
|  | R | 5′-aacaccttctgctgcgtctc-3′ |
| SUMO1 | F | 5′-aatccaccgacaccatgtct-3′ |
|  | R | 5′-tggatctcactgctgtcctg-3′ |
| β-actin | F | 5′-tgggacgacatggagaaaa-3′ |
|  | R | 5′-ctggaaggtggacagcgag-3′ |


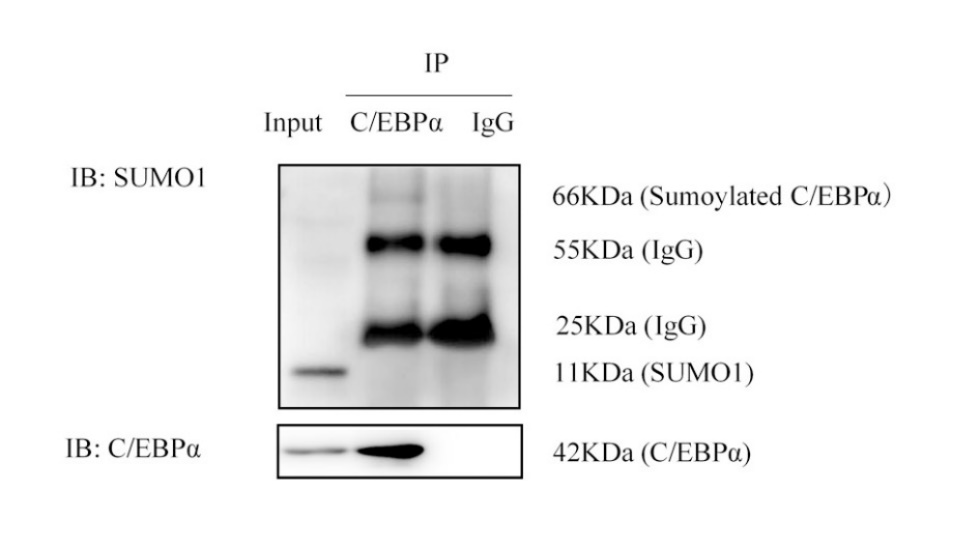


**Supplementary Figure S1: Co-Immunoprecipitation (CO-IP) on rat lung tissues to prove the hypothesis of C/EBPα sumoylation.** Samples were derived from the normoxia group rats at postnatal day 7 (P7).

**
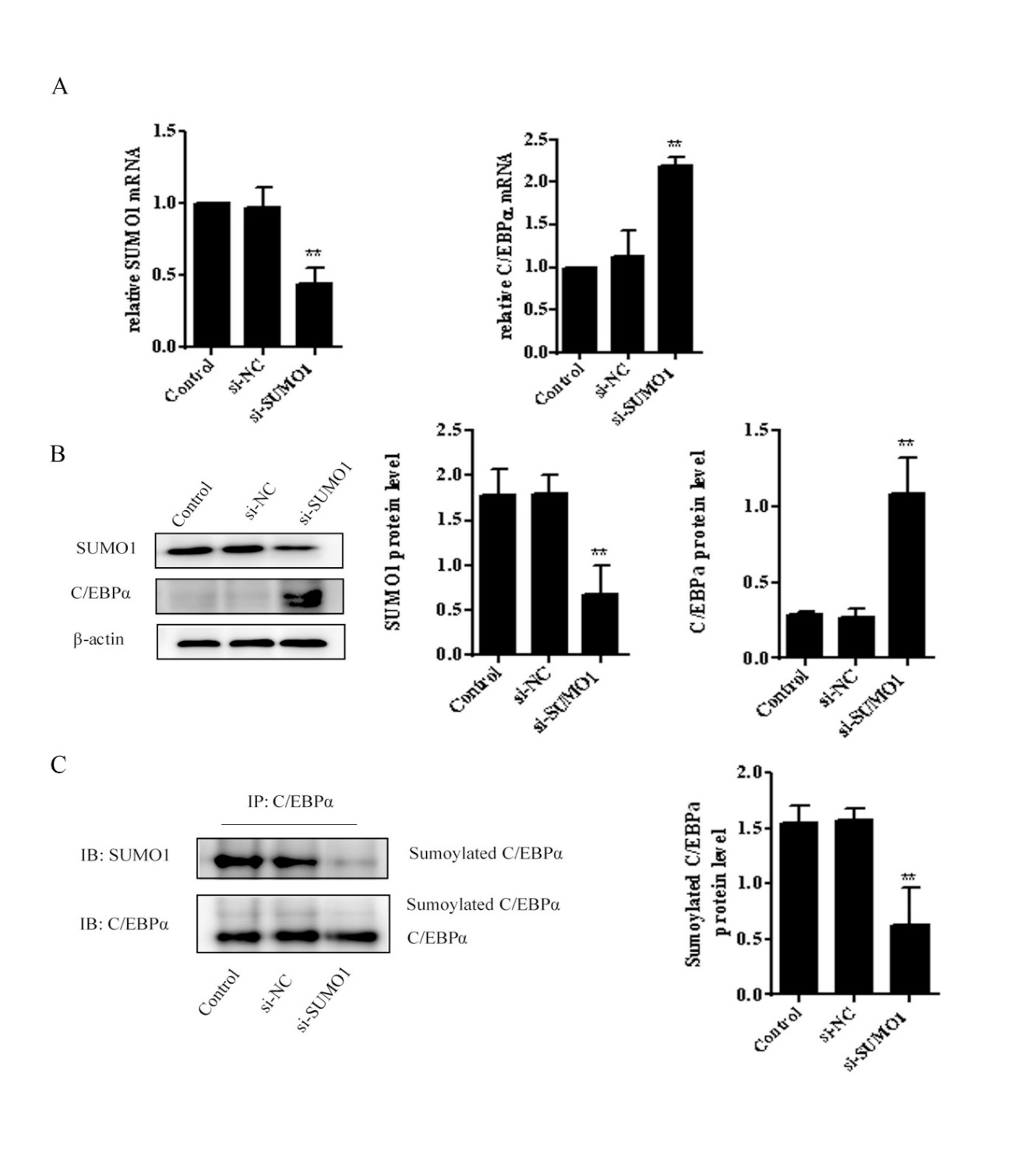
**

**Supplementary Figure S2:**  ***SUMO1* knockdown reduced the levels of SUMO1 and sumoylated C/EBPα, while increased C/EBPα expression in the lungs of BPD rats.**  Three neonatal rats per group were selected were sacrificed from newborn rats at P14 and one sample repeated three times in our experiment. RT-PCR and western blot analysis of SUMO1 and C/EBPα mRNA and protein in rat lungs. β-actin was used as the loading control (A and B). The levels of co-Immunoprecipitated sumoylated C/EBPα after transfection of newborn rat lungs with *SUMO1*-RNAi-NC-LV (C). The data shown were acquired from at least three independent experiments and values represent mean ± SD; ** *P* < 0.05 vs control.


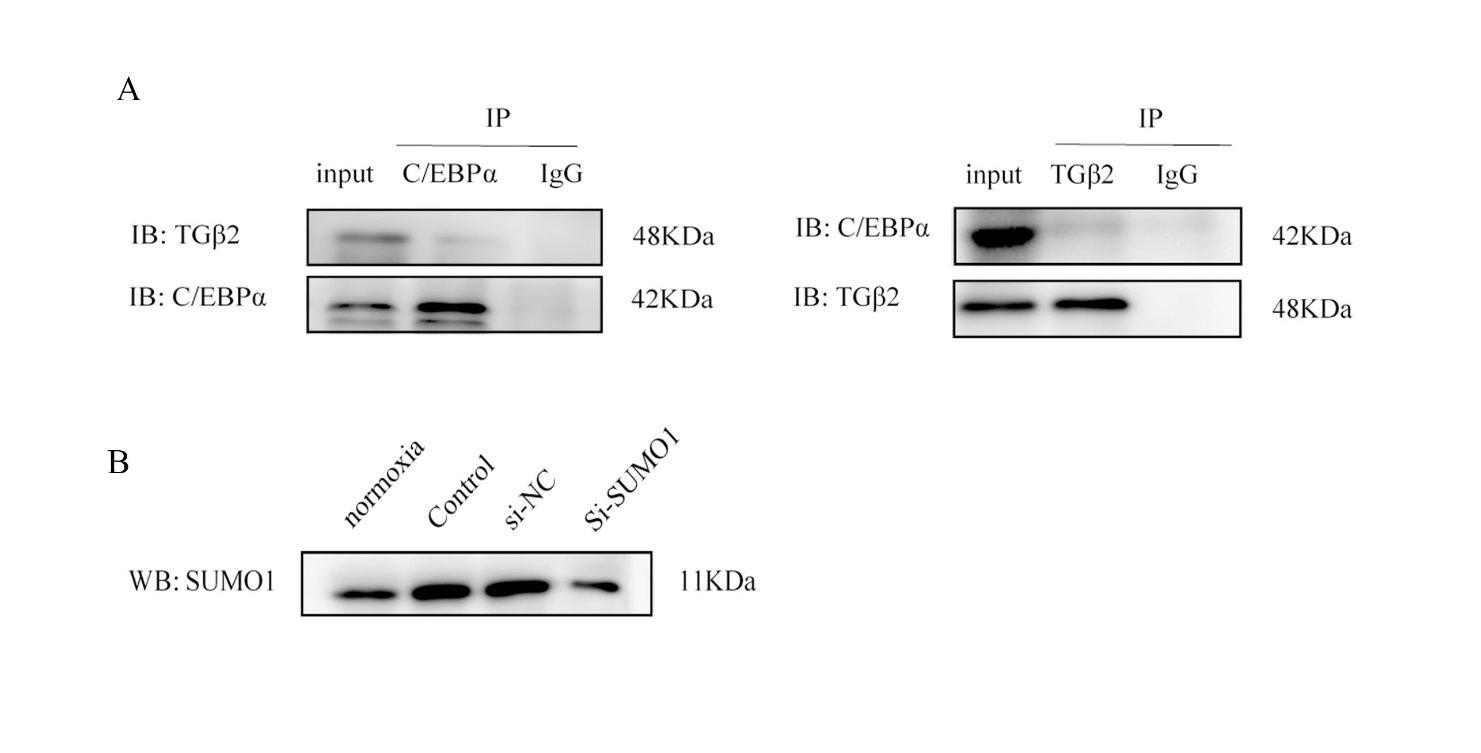


**Supplementary Figure S3:** The interaction between C/EBPα and TGFβ2 observed by Co-IP assay in lung (A). Western blot of SUMO1 after transfection of newborn rat lungs with *SUMO1*-RNAi-NC-LV (B). Three neonatal rats per group were selected were sacrificed from newborn rats at P14 and one sample repeated three times in our experiment.
